# Blood-Based mRNA Tests as Emerging Diagnostic Tools for Personalised Medicine in Breast Cancer

**DOI:** 10.3390/cancers15041087

**Published:** 2023-02-08

**Authors:** Helena Čelešnik, Uroš Potočnik

**Affiliations:** 1 Faculty of Chemistry and Chemical Engineering, University of Maribor, Smetanova Ulica 17, 2000 Maribor, Slovenia; 2 Center for Human Genetics & Pharmacogenomics, Faculty of Medicine, University of Maribor, Taborska Ulica 8, 2000 Maribor, Slovenia; 3 Department for Science and Research, University Medical Centre Maribor, Ljubljanska Ulica 5, 2000 Maribor, Slovenia

**Keywords:** breast cancer, peripheral blood, diagnostic assay, mRNA test, transcriptome, early detection, cancer screening

## Abstract

**Simple Summary:**

Molecular diagnostic tests can facilitate molecular classification of breast cancer and its clinical management, including prediction, prognosis, and selection of therapy. Messenger RNA tests can supplement the clinically widely used DNA genetic testing. For instance, they can offer valuable information beyond DNA in cases where DNA variants are difficult to interpret. Moreover, several tissue-based mRNA tests for breast cancer are already used routinely in clinical practice to assess the recurrence risk and guide adjuvant endocrine therapy and chemotherapy. Here, we bring attention to the recently developed and commercially available blood mRNA diagnostic tests for breast cancer, which offer several advantages over tissue-based tests, including minimal invasiveness, absence of heterogeneity problems, cost-efficiency, and possibilities for early cancer detection well before the currently used conventional diagnostic approaches. We also investigate the state-of-the-art blood transcriptomic breast cancer research aimed at bringing novel blood transcriptome biomarkers and next-generation sequencing technology into clinical practice.

**Abstract:**

Molecular diagnostic tests help clinicians understand the underlying biological mechanisms of their patients’ breast cancer (BC) and facilitate clinical management. Several tissue-based mRNA tests are used routinely in clinical practice, particularly for assessing the BC recurrence risk, which can guide treatment decisions. However, blood-based mRNA assays have only recently started to emerge. This review explores the commercially available blood mRNA diagnostic assays for BC. These tests enable differentiation of BC from non-BC subjects (Syantra DX, BCtect), detection of small tumours <10 mm (early BC detection) (Syantra DX), detection of different cancers (including BC) from a single blood sample (multi-cancer blood test Aristotle), detection of BC in premenopausal and postmenopausal women and those with high breast density (Syantra DX), and improvement of diagnostic outcomes of DNA testing (variant interpretation) (+RNAinsight). The review also evaluates ongoing transcriptomic research on exciting possibilities for future assays, including blood transcriptome analyses aimed at differentiating lymph node positive and negative BC, distinguishing BC and benign breast disease, detecting ductal carcinoma in situ, and improving early detection further (expression changes can be detected in blood up to eight years before diagnosing BC using conventional approaches, while future metastatic and non-metastatic BC can be distinguished two years before BC diagnosis).

## 1. Introduction

Breast cancer (BC) is the most diagnosed cancer in women, and among the leading causes of cancer mortality. It is estimated that over 86,000 European women will die of this disease in 2022 [1,2]. BC is a heterogeneous cancer comprised of distinct subtypes with specific pathological characteristics and clinical implications. Based on the expression of the estrogen receptor (ER), progesterone receptor (PR), and the amplification of the HER2 (ERBB2) receptor, BC is categorised into four *clinical* subtypes: *hormone receptor (HR)-positive* (ER+/PR+/HER2−), *triple positive* (ER+/PR+/HER2+), *HER2-positive* (ER−/PR−/HER2+), and *triple-negative breast cancer* (TNBC) (ER−/PR−/HER2−, also HR−/HER2−) [3,4]. This classification is clinically important, because it helps to assess cancer prognosis, and, in company with other traditional prognostic and predictive factors, select an appropriate treatment strategy. ER-positive tumours respond to drugs that target the ER pathway, such as the selective ER modulators and ER degraders (tamoxifen, fulvestrant) or aromatase inhibitors (letrozole, anastrozole, exemestane) [5]. Tumours with HER2 amplification respond to anti-HER2 therapies (i.e., monoclonal antibodies targeting HER2, such as trastuzumab and pertuzumab) [6]. However, TNBC does not express ER, PR, and lacks HER2 amplification, and therefore targeted therapies are lacking for this subtype, since targeted therapies are commonly aimed at these receptors. Consequently, TNBC represents a major clinical challenge, and is associated with a poor prognosis connected with metastasis, post-treatment relapse, and reduced survival. While the standard treatment for TNBC involves chemotherapy, new immunotherapy options have become available in recent years [7,8]. One important characteristic of TNBC is its high mutational burden [9,10], which is associated with tumour immunogenicity, and may aid in the selection of TNBC patients likely to benefit from certain immunotherapies [11]. Treatment decisions for this heterogeneous cancer may also be guided by RNA-based molecular subtyping. The web-based TNBCtype algorithm was developed as an online tool to distinguish six TNBC subtypes with different biological characteristics and therapy options [12,13].

In addition to the clinical subtyping of BC, a detailed molecular characterisation of BC tissues, which employed DNA analyses (DNA mutations, copy number, DNA methylation), mRNA profiling and protein expression of tumour tissues, has led to the classification of four *molecular/intrinsic* BC subtypes with different prognoses and survival: *Luminal A* (ER+/PR+/HER2−, low proliferation factor Ki67+, low grade), *Luminal B* (ER+/PR±/HER2±, high Ki67+ (≥14%), high grade), *HER2-enriched* (ER−/PR−/HER2+, high proliferation, any Ki67 level), and *basal-like* (ER−/PR−/HER2−, high proliferation, any Ki67 level, high grade, necrosis) [3,14]. Intrinsic and clinical subtypes overlap to some degree, but not completely. For instance, basal-like breast cancers are mostly enriched in TNBC. However, about 21% of TNBCs are not basal-like [15].

Molecular diagnostic tests for BC are a form of precision medicine that uses genetic, genomic, or other molecular methods to help clinicians learn about the characteristics and underlying biological mechanisms of their patients’ breast cancer. These tests rely increasingly on advanced high-throughput methodologies (for instance, next-generation sequencing—NGS), and can be used for molecular subtyping, prediction, prognosis, and choice of therapy, thereby facilitating health care decisions.

## 2. Molecular Diagnostic Tests and Companion Diagnostic Devices for Breast Cancer Approved by the US Food and Drug Administration (FDA)

The diagnostic tests available for BC are intended to detect variations in DNA sequence, analyse RNA profiles, or measure protein expression, in order to determine the genetic carrier status, classify BC and its subtypes, detect cancer spreading, facilitate prognosis, and/or assess recurrence risk. In clinical care, a number of *nucleic acid-based tests* have been adopted for BC in recent years. Some of them have been cleared or approved by the FDA (Supplementary file S1, Table S1) [16]. These include multigene expression assays, tests for variant detection in BRCA1, BRCA2, PIK3CA, and Topoisomerase II Alpha genes, and assays for determination of HER2 status. In addition, a number of *companion diagnostic devices* for BC have been approved by the FDA (Supplementary file S1, Table S2) [16]. These are in vitro diagnostic (IVD) devices and imaging tools that provide information needed for the safe and effective use of corresponding therapeutics. For BC, FDA-approved IVD devices include testing for BRCA 1, BRCA2, and PIK3CA mutations, HER2 gene amplification, and protein expression of HER-2, PD-L1, and Ki67 (Supplementary file S1, Table S2). These DNA and protein-level tests facilitate decisions on therapy with Herceptin, Kadcyla, Perjeta, Lynparza, Talzenna, Piqray, Verzenio, and Keytruda. Some tests are classified both as a nucleic acid-based test and IVDs.

## 3. Cancer Diagnostics Based on mRNA Can Offer Prognostically Useful Information beyond DNA Variation

Messenger RNA diagnostics are able to provide important information that cannot be learned directly from mutation data [17]. For instance, although evaluating a predisposition to hereditary cancer using DNA genetic testing is becoming widespread, it can often be challenging to interpret the detected variants accurately, especially when DNA alterations are predicted to impact splicing. In these cases, the variants are frequently classified as uncertain, or likely pathogenic. However, RNA testing can help interpret the impact of these changes by enabling their classification as pathogenic/clinically actionable or benign [18], thereby informing clinical decisions. According to Karam et al., blood RNA testing provided as a supplement to DNA genetic testing has the potential to affect medical management in at least 1 in 43 patients [18].

Additionally, although hereditary BC risk has been linked to highly penetrant genetic variants, many of their carriers never develop BC, and many BC patients do not carry these variants [19,20]. This can create uncertainty for both the patients and the physicians when it comes to making decisions whether to pursue aggressive prevention strategies (e.g., prophylactic surgery, chemopreventive intervention). To get a more accurate assessment of individual risk, examining gene-expression patterns can make it easier to differentiate individuals from high-risk families who are likely to develop BC from those who are not [21]. This way, women who carry known susceptibility variants can be assigned different individual risks based on their gene expression profiles, which may facilitate personalised prevention decisions [21].

The clinical contribution of mRNA testing beyond that of DNA becomes particularly apparent in early-stage BC diagnosis and assessment of BC recurrence risk, which can guide treatment decisions [17]. For example, the 21-gene expression tissue assay Oncotype DX can identify pathway changes on a very detailed level, and predict recurrence within the HR-positive BC more precisely than any DNA-based assay [17,22]. While genomic mutations can lead to changes in the downstream pathways, there may be several intermediate steps between the driver mutations and the disease phenotype. However, distinct expression patterns that can result from the driver mutations can be identified easily by examining the mRNA levels. Consequently, mRNA analysis can be more informative than analysis of genomic variation.

While several *tissue-based* mRNA assays are used and/or FDA-approved (Supplementary file S1, Table S1), only a few *blood-based* mRNA tests (described below) are commercially available, and none have so far been approved by the FDA. Additionally, none of the current FDA-approved companion devices for BC are based on mRNA detection, either in blood or in tissues (Supplementary file S1, Table S2).

## 4. Tissue-Based mRNA Expression Assays for Breast Cancer

Tissue gene expression assays investigate the patterns of a selected number of different genes in cancer cells obtained during surgery or biopsy, to help inform clinical decisions (Table 1). For instance, several multigene prognostic assays (e.g., Oncotype DX, Prosigna, EndoPredict), enable estimation of the residual risk of recurrence following surgery. This can guide clinical decisions, such as whether chemotherapy or other treatments are needed to reduce risk after surgery. Consequently, this can facilitate safe avoidance of patient over-treatment.

### 4.1. Prosigna Breast Cancer Prognostic Gene Signature Assay (Formerly Called the PAM50 Test)

Prosigna (Veracyte, San Francisco, CA) is an FDA-approved test that uses a 50-gene expression profile of BC tumour tissue to inform prognostic, recurrence and therapeutic management of BC [23,24,25,26]. It is intended for post-menopausal women with stage I or II, lymph node (LN) negative or positive (1–3 positive LNs), hormone-receptor (HR)-positive BC, with a tumour size of <5.0 cm. Prosigna can help assign BC to one of four intrinsic subtypes and provide a risk assessment for distant recurrence at ten years. The results of this test, together with other clinical information, can be used by physicians for assessing the recurrence risk. Based on the low, intermediate or high risk of distant recurrence, physicians can decide if patients can safely avoid adjuvant chemotherapy [25,27].

### 4.2. MammaPrint Test (also Called the 70-Gene Signature)

MammaPrint (Agendia, Inc., Amsterdam, The Netherlands) is an FDA-cleared prognostic assay, which analyses the expression of 70 genes to help predict if BC will spread to other parts of the body or return [28,29,30]. Based on the calculated recurrence score, women are categorised into a high or low risk group, which can guide decisions on adjuvant endocrine and chemotherapy [28,31]. MammaPrint is performed on fresh or preserved tissues from biopsy or surgery, and is intended for early-stage invasive HR-positive or HR-negative, stage I or II cancers, smaller than 5.0 cm, present in three or fewer lymph nodes [32]. It can be integrated into diagnostic workups for quicker, more informed decisions on pre- and post-operative treatment. BluePrint, another assay by Agendia, Inc., is a molecular classification system that analyses 80 genes to identify the underlying biology of individual breast cancers. It enables BC subtyping into luminal type (luminal A or luminal B), HER2-type, and basal-like type. This classification reveals valuable information about long-term prognosis and response to systemic therapy, and can enable patient selection for either chemotherapy or endocrine treatment [33].

### 4.3. Oncotype DX Breast Recurrence Score Test

Oncotype DX (Genomic Health, Redwood City, CA, USA) [34] is a gene expression profiling assay intended for HR+/HER2− BC, stage I, II or IIIa, LN-negative, or up to three positive LNs. It analyses the expression of 21 genes in cancer cells obtained by biopsy or surgery, and helps assess the risk of BC recurrence. The calculated recurrence score enables an individualised estimate of 9-year distant recurrence risk and the likelihood of adjuvant chemotherapy benefit [35,36]. Oncotype DX became commercially available in 2004, and, as a laboratory-developed test, did not require FDA-approval [37]. The assay is included in international treatment guidelines, and is performed at a CLIA-certified central laboratory in the USA. It has been validated extensively in clinical studies. A large adjuvant BC treatment clinical trial (TAILORx, NCT00310180), which included 10,253 patients worldwide, demonstrated that, by using Oncotype DX, it is possible to identify BC patients who may not need chemotherapy to increase their chance of survival [37,38,39,40,41].

### 4.4. Breast Cancer Index, BCI

BCI (Biotheranostics, Inc., San Diego, CA, USA) is a predictive multi-gene expression assay that incorporates: (1) the HOXB13:IL17BR ratio, which is associated with tumour responsiveness to endocrine therapy; and (2) the molecular grade index, which is associated with tumour proliferation, and is based on the average expression of five cell cycle-associated genes [42,43]. BCI helps to predict the risk of early-stage HR+ BC coming back 5 to 10 years after diagnosis. According to the American Society of Clinical Oncology (ASCO) guidelines and NCCN Clinical Practice Guidelines in Oncology (NCCN Guidelines^®^), this assay can also help physicians decide if extending hormonal therapy in patients with HR+/HER2−, LN-negative or LN-positive (1–3 nodes) BC beyond five years (for a total of 10 years) would be beneficial [31]. The clinical validation and the indication of prognostic utility for this molecular signature was provided by the TransATAC and the Stockholm trials [42,44].

### 4.5. EndoPredict Breast Cancer Prognostic Test

EndoPredict (Myriad Genetics, Salt Lake City, UT, USA) is a gene expression test for patients with early-stage, ER+/HER2− BC. It analyses 12 genes related to tumour proliferation and hormone receptor activity. It calculates a risk score, which can be used together with clinical pathological factors (such as tumour size and nodal status) to guide treatment decisions for chemotherapy and extended endocrine therapy [45,46]. It enables prediction of early and up to 15 years distant recurrence (metastatic disease) [47,48,49]. It has been validated clinically in both post- and pre-menopausal patients [50,51]. By using EndoPredict, it was possible to identify 65% of premenopausal patients with low-risk disease who could safely forgo adjuvant chemotherapy (independent from nodal status) [51].

### 4.6. GeneSearch Breast Lymph Node (BLN) Test Kit

GeneSearch BLN (Veridex, LLC., Raritan, NJ, USA) is an FDA-approved gene expression test for patients with invasive BC, intended to detect whether BC cells have spread to lymph nodes under the arm [52]. The assessment is based on the analysis of two gene expression markers, mammaglobin (MG) and cytokeratin 19 (CK19) in lymph node(s) removed during surgery. These mRNA markers are expressed at high levels in breast tissue, but only at low levels in normal lymph node tissue (i.e., cell-type-specific mRNAs) [53,54,55,56]. Metastases greater than 0.2 mm can be detected by the BLN Test in nodal tissue removed from sentinel lymph node biopsies, and the assay results can be used to guide the decision to remove additional lymph nodes [52].

**Table 1:** Tissue mRNA-level diagnostic tests for breast cancer.

Assay Trade Name (Manufacturer)	Number of Genes; Sample Type	Assay Indicated For	Description	Methodology/ Platform	FDA Numbers; References
Prosigna Breast Cancer Prognostic Gene Signature Assay (Veracyte, Inc.)	50 genes; BC tissue (formalin-fixed paraffin embedded—FFPE)	HR+, LN-negative or 1–3 positive nodes, stage I or II cancers	Classification of intrinsic BC subtypes; prognostic; recurrence risk assessment; guides adjuvant endocrine and chemotherapy in postmenopausal patients	nCounter Dx Analysis System (mRNA hybridization to DNA probes)	FDA (K130010); [26,31]
MammaPrint (Agendia, Inc.)	70 genes; BC tissue (FFPE or fresh)	HR+ or HR−, LN-negative or 1–3 positive nodes, stage I or II cancers, ≤5.0 cm	Prognostic; recurrence risk assessment; guides adjuvant endocrine and chemotherapy in patients >50 or postmenopausal patients	Microarray-based assay; also available as a targeted RNA next-generation sequencing assay	FDA (K101454, K081092, K080252, K070675); [31,57,58]
Oncotype DX Breast Recurrence Score Test (Genomic Health)	21 genes (16 cancer-related and 5 reference genes); BC tissue (FFPE)	HR+/HER2−, LN-negative or 1–3 positive nodes, stage I, II or IIIa cancers, ≤5.0 cm	Prognosticates distant recurrence; predicts chemotherapy benefit/guides adjuvant endocrine and chemotherapy in postmenopausal or premenopausal patients	qRT-PCR-based assay	[31,34,35,36,59]
Breast Cancer Index—BCI (Biotheranostic, Inc.)	7 genes; BC tissue (FFPE)	HR+, LN-negative or 1–3 positive nodes, stage I–III cancers, invasive BC cases without evidence of recurrence	Prognosticates risk of distant recurrence; predicts likelihood of benefit from extended (>5 years) endocrine therapy; guides adjuvant endocrine and chemotherapy in patients >50 or postmenopausal patients	qRT-PCR-based assay	[31,60]
EndoPredict (Myriad Genetics)	12 genes (8 BC-related and 4 reference genes); BC tissue (FFPE)	HR+/HER2−, LN-negative or 1–3 positive nodes, tumour size T1–T3, grade 1–3	Predicts distant recurrence at 10 years (and up to 15 years); guides adjuvant endocrine and chemotherapy in patients >50 or postmenopausal patients; identifies premenopausal patients who do not need chemotherapy	qRT-PCR-based assay	[31,46,61]
GeneSearch Breast Lymph Node (BLN) Test Kit (Veridex, LLC.)	3 genes (2 metastasis-related and 1 reference); lymph node(s) removed during surgery	Patients with invasive BC, scheduled for sentinel lymph node dissection	BC metastasis detection	qRT-PCR-based assay	FDA: P060017 S001–S004, [52]

## 5. The Advantages of Using Peripheral Blood (i.e., a Blood-Based Liquid Biopsy) for Cancer Diagnostics

Cancer biomarker research has concentrated largely on the molecular characteristics of tumour cells, and on immune responses in the tumour microenvironment, leading to the emergence of tissue-based diagnostic assays. However, focusing on blood biomarkers has many advantages for cancer screening, diagnosis, monitoring, and prognosis.

First, drawing peripheral blood offers simple, cost-effective and minimally invasive sampling. Additionally, it is well known that patient survival rates are greatly increased if cancer is identified at its early stages [62], and several lines of evidence indicate that analysing blood allows the detection of very early systemic changes, which is crucial for cancer screening [63,64,65,66]. On the other hand, biopsies may not be appropriate for cancer screening, and may deter healthy people with no symptoms [67]. Currently, almost 50% of cancers are diagnosed at the later stages, when the symptoms begin to manifest. At that time, the disease outcomes are poorer and the treatments more expensive [68]. The less invasive blood sampling may make early cancer detection and accompanying medical intervention more feasible. A liquid biopsy may also offer advantages compared to the existing *imaging* methods. About half of women have dense breast tissue, which is associated with reduced sensitivity on mammography [69]. Additionally, the tumour has to reach a certain size to be detectable by imaging [70]. Moreover, although close to 20% of patients with invasive BC are women under 50, in some countries, women in this age group are not typically referred for mammography screenings [71]. A liquid biopsy could represent a solution to these issues.

Although repeated tissue biopsies can be useful in clinical practice to help monitor how the tumour evolves, tissue collection comes with risk of complications [66]. It may also provide insufficient material, and can have selection bias due to tumour heterogeneity (e.g., single-site tissue biopsies may give only a snapshot of the tumour) [72]. In contrast, peripheral blood is readily available, and not prone to selection bias or heterogeneity problems.

Since blood testing has the potential to circumvent all the above-mentioned disadvantages of biopsies and imaging, liquid biopsy has been gaining considerable interest in BC precision medicine. A variety of biomarkers with clinically useful information can be detected in blood, such as circulating tumour cells (CTCs), circulating tumour-derived material (e.g., circulating tumour DNA—ctDNA), extracellular vesicles, cell-free microRNAs (cfmiRNAs), methylation markers, and others. These have been reviewed in several excellent papers, e.g., [73,74,75,76,77]. Here, we will focus on mRNA biomarkers from peripheral blood cells and their use in cancer diagnostics.

## 6. Research Focusing on BC-Specific Transcriptional Profiles in Peripheral Blood Has Established Their Diagnostic and Prognostic Value

During cancer, the proportion of effector and regulatory immune cells and their gene expression profiles change in the tumour microenvironment. Similar immune changes can be detected in peripheral blood cells, since the circulatory system participates in physiological and pathological activities throughout the body [78,79]. Tumours release a variety of signalling molecules that are detected by the circulating blood cells. These respond with phenotypic and functional changes that enable them to modulate the cancer’s progression. This can result either in the killing of tumour cells and cancer rejection, or the promotion of cancer proliferation and spreading [80,81,82].

In recent years, high-throughput transcriptomic studies have revealed meaningful differences in peripheral blood cells between BC and healthy subjects, and within BC patient subpopulations. Specific transcriptome changes have been identified with clinical utility for BC patients, indicating a clear potential for liquid biopsy and the development of *blood-based messenger RNA* diagnostic tests [83,84,85].

### 6.1. Identification of Blood Predictor Genes That Can Distinguish BC Patients from Other Cancer Patients and Healthy Subjects

The transcriptome of circulating blood cells is markedly perturbed by the presence of a breast carcinoma [4]. Aarøe et al. identified a blood gene expression signature that distinguished BC patients and healthy women with a prediction accuracy of 79.5%, a sensitivity of 80.6%, and a specificity of 78.3% [84]. Microarray analysis was performed on whole blood samples from 121 females who had had an initial suspicious screening mammogram (of those, 67 had BC and 54 had no malignant disease), and 6 healthy controls. The deregulated genes in BC patients were linked to defence responses, translation, and various metabolic processes (e.g., lipid metabolism). Furthermore, using a microarray analysis of peripheral blood cells, Dumeaux et al. constructed a 50-gene signature, characterised mainly by genes associated with systemic immunosuppression, that could indicate the presence of BC, and classify women with changes other than BC (i.e., population-based controls, gastrointestinal and brain cancer patients) as negative [86]. RNA sequencing was employed by Suzuki et al. to analyse peripheral blood mononuclear cells (PBMCs) from 13 BC patients and 3 healthy volunteers [87]. The PBMC transcriptome identified a significant unique gene expression signature in BC patients, which involved upregulation of immune response-related pathways: TLR3- and TLR4-induced TICAM1-specific signalling pathways, and the Th17, Th22, and Th9 cell differentiation pathways.

Ideally, a reliable diagnostic tool for early BC detection in peripheral blood would achieve high prediction accuracy with as few genes as possible, since this would reduce the required examination of a large number of genes and associated computational complexity, and lower the clinical cost of diagnosis [88]. This was recognised by Zhang et al., who reported a five-marker panel for early detection of BC in peripheral blood. The markers in the panel were associated with signalling, steroid hormones, metabolism, immune system and hemostasis. They also showed how to use an SVM (support vector machine) to model the classification and prediction problem of early BC detection in peripheral blood [88].

To address the need for early biomarkers that could be useful for women with BC who have no symptoms, a 2020 study by Hou et al. identified a 10-gene expression signature that differentiated healthy subjects from patients with breast lesions (ranging from high-risk *benign* to *malignant* breast lesions) with 100% sensitivity, 84.2% specificity and 93.5% accuracy [89]. The identified signature biomarkers were involved in apoptosis, the immune system, gene transcription, and post-transcriptional protein modification. Microarray analysis was performed using whole blood of 57 BC patients, 50 patients with high-risk benign breast disease, and 44 healthy subjects.

There is substantial interest in universal cancer screening, where a single test can be used for multiple cancers [90]. Recent research published by Qi et al. in 2021 focused on non-invasive blood-based transcriptome analysis that can identify multiple cancers simultaneously, and can, thus, be used for pan-cancer diagnostics [91]. RNA sequencing of whole blood from 30 healthy individuals and 45 cancer patients (with cancer of the breast, oesophagus, stomach, thyroid, rectum, colon, and uterus) indicated that, in addition to mRNAs, long non-coding (lnc) RNAs and very long intergenic non-coding (vlinc) RNAs can also serve as quality biomarkers. Interestingly, the authors observed that, to some extent, normal ageing and cancer have somewhat similar effects on blood transcriptomes. However, there are also remarkable differences, with a cancer transcriptome dominated by specific upregulation of immune and stress-related functions.

Tumour-educated blood platelets (TEPs) can provide another valuable platform for pan-cancer screening and companion diagnostics [92]. These cells are involved in systemic and local responses to tumour growth. In 2021, an RNA sequencing study of 283 platelet samples by Best et al. revealed that their transcriptome differences were able to distinguish 228 patients with localised and metastasised tumours and 55 healthy individuals with 96% accuracy [93]. Notably, the correct location of the primary tumour was identified with 71% accuracy across six different tumour types: non-small cell lung carcinoma, colorectal cancer, glioblastoma, pancreatic cancer, hepatobiliary cancer, and BC.

### 6.2. Identification of Expression Signatures That Can Distinguish between Breast Cancer and Benign Breast Disease

Yang et al. reported that peripheral blood transcriptome profiles can differentiate between BC and benign breast disease in non-conclusive mammography patients. They examined microarray data from the whole blood of 84 BC and 94 benign breast disease patients, and described a 28-gene expression signature that could distinguish these groups. They reported a rather high false positive rate of 29% and false negative rate of 20% in the BI-RADS 0 group [94]. The 28-gene panel included genes involved in immune response, transcription regulation, apoptosis, and breast disease-related genes. The expression of immune response genes was lower in BC than in benign breast disease.

### 6.3. Identification of Blood Expression Signatures That Can Distinguish between Lymph-Node Positive and Negative BC

Zuckerman et al. [95] used microarray analysis to identify gene expression patterns in PBMCs from patients with node-positive (LN+) and node-negative (LN−) BC. They observed that expression levels of immune-related genes (e.g., lymphocyte activation and B-cell-related pathways) were downregulated, while tumour-promoting pathways were upregulated in the peripheral blood of LN+ compared to LN− patients. It is known that tumours can modulate the host immune responses, which enables cancer development, overall cancer progression, escape, and metastasis. The authors suggested that, compared to LN+ patients, the host immune responses in LN− patients were more intact, and thereby could resist tumour-mediated immune modulation and associated tumour invasion.

### 6.4. Blood Gene Expression Alterations Years before BC Diagnosis

Prospectively collected blood samples (i.e., acquired before BC diagnosis) from the Norwegian Women and Cancer (NOWAC) cohort have offered an insight into the diagnostic potential that blood gene expression can offer years before diagnosis. The use of new approaches for statistical analyses of time-dependent curves of gene expression levels helped reveal that gene expression profiles in blood cells differ between future BC cases and healthy subjects up to eight years before diagnosis [65,96,97].

Additionally, potentially wide-reaching differences in blood gene expression between metastatic and non-metastatic BC cases can be observed up to two years before diagnosis, as reported in 2020 by Holsbø and Olsen [64]. They used Illumina bead chips to analyse gene expression in whole blood taken from 197 subjects before they were diagnosed with breast cancer, which were compared with 197 age-matched controls. Plasmacytoid dendritic cell function, the SLC22 family of transporters, and glutamine metabolism were identified as potential links between the immune system and metastasis [64].

### 6.5. Peripheral Blood Transcriptomics May Open the Way to Novel Immune BC Subtyping

BC is not a single homogeneous disease. Rather, it consists of multiple subtypes, each with a distinct clinical progression. Several reports have investigated the possibility of classifying BC subtypes based on gene expression in blood [4,98,99,100,101]. Their findings revealed that peripheral blood transcriptomes of BC patients correlate poorly with classical BC subtypes [87,101,102]. This is not surprising, considering that gene expression in peripheral blood cells represents the systemic immune reaction to the presence of tumour cells. Therefore, it is generally not expected to mirror the established BC subtypes, which are categorised according to the expression of ER, PR, and HER2 receptors on the surface of tumour cells [4]. In a 2019 study by Ming et al., the use of unsupervised cluster analysis of PBMC transcriptome data from BC patients revealed two new BC subgroups. They differed by distinctive immune responses to a tumour, including immune cell activation, regulation of the innate and adaptive immune system, antibody production, and inflammation level. Cancer patients with all the classical BC subtypes were represented in both groups [101]. Similarly, RNA sequencing of PBMCs from BC patients by Suzuki et al. in a 2019 study revealed a segregation of BC blood transcriptomes into two distinct subsets that did not correlate with the classical BC subtypes. Their difference was primarily in B-cell receptor immunological pathways and chemoattractant receptor-homologous molecules on Th2 (CRTH2) signalling in Th2 cells [87]. The evidence thus indicates that the PBMC transcriptome in BC patients is affected by the presence of cancer, not by BC subtypes [87,101]. Based on these findings, a new—PBMC transcriptome-based—subtyping was proposed by Ming et al. as an independent classification for BC patients [101]. This subtyping could have implications for personalised BC management, for example, for cancer prognosis, treatment monitoring, and the selection of patients for immunotherapy [4].

## 7. Commercially Available Blood-Based mRNA Tests for Breast Cancer

Currently, blood analysis in clinical management of BC is aimed mostly at detecting DNA-level and protein-level biomarkers. Examples include the BRCA1 and BRCA2 genes, which are the classical blood-based biomarkers for genetic screening of individuals with hereditary BC susceptibility [103]. Other blood BC biomarkers include proteins that can help assess disease progression, predict recurrence, and facilitate the monitoring of treatment response, such as carcinoembryonic antigen (CEA), gene products of MUC1 (e.g., cancer antigen (CA) 15-3 and CA 27.29), circulating cytokeratins, and serum HER2 [104,105].

However, recent years have brought a gradual emergence of mRNA-level blood diagnostics, with new mRNA assays finding their way increasingly into the clinical setting (Table 2). As detailed below, these blood tests can facilitate healthcare decisions in several ways; by supplementing hereditary DNA-testing for better diagnostic outcome (i.e., helping to classify DNA variants) (+RNAinsight), by enabling early BC detection (Syantra DX Breast Cancer, BCtect), and by facilitating simultaneous screening of several cancers (multi-cancer blood test Aristotle).

### 7.1. Hereditary Breast Cancer Predisposition

To improve the diagnostic outcome of blood- or salivary-DNA genetic testing in patients assessed for hereditary cancer predisposition, in 2020, Ambry Genetics (Aliso Viejo, CA, USA) added whole blood RNA genetic testing (i.e., ***+RNAinsight***) to their available DNA-level genetics hereditary cancer panels [18,106,113,114]. First, the inherited risk for several types of cancers (breast, ovarian, prostate, colon, pancreatic, uterine, and others) is identified simultaneously by analysing the genes in the hereditary cancer panels. The panels employ next-generation sequencing (NGS), or Sanger sequencing, of all coding domains and the flanking 5′ and 3′ ends of all introns and untranslated regions, with confirmatory PCR, multiplex ligation-dependent probe amplification (MLPA), and/or targeted chromosomal microarray analyses. Then, whole-blood RNA testing (+RNAinsight) by RNA sequencing of up to 91 genes is added to help classify DNA variants associated with cancer. This provides functional RNA information that can help identify and interpret DNA variants, including deep intronic variants.

### 7.2. Breast Cancer Screening

The ***Syantra DX Breast Cancer test*** (Syantra Inc., NW Calgary, Canada) is a BC screening assay that uses whole blood to measure the gene expression characteristics of a 12-gene biomarker panel. Quantitative PCR expression data are analysed with a Syantra software package developed with machine learning, in order to interpret the results and classify the samples as positive or negative for the BC signature [107,108]. A prospective blinded international study (NCT04495244) is currently ongoing, and enrolling women from 30 to 75 years of age with a BI-RADs 3–5 score on a screening mammogram, or (for the controls) a normal screening mammogram or physical exam. The interim results have been made available in 2022, and have demonstrated an inferred accuracy of 92%, a specificity of 94%, and a sensitivity of 79% for BC detection [115]. Small tumours less than 10 mm were detected with a sensitivity of 68.4%, thereby demonstrating the clinical utility of the Syantra DX Breast Cancer test for early screening. This test also shows strong performance for women under 50 and those with high breast density [115]. It can detect the presence of the most common types of invasive BC, but has not been studied for inflammatory BC. Research is ongoing for the detection of ductal carcinoma in situ (DCIS) and additional applications. The test is currently provided in accredited laboratories across Canada.

The ***multi-cancer blood test Aristotle*** (Stage Zero Life Sciences Ltd. Richmond Hill, Ontario, Canada) [109,116] uses mRNA expression in whole peripheral blood for simultaneous early detection of multiple cancer signatures from a single blood sample. These include cancers of the breast, bladder, colorectum, cervix, endometrium, liver, ovary, prostate, and stomach. High sensitivity and specificity were observed for each cancer in a 2020 report; for instance, BC was classified with a sensitivity of 87.2% at 99.0% specificity (n = 94) [116]. Aristotle was developed with logistic regression using Affymetrix gene expression profiles generated from 2845 unique human whole peripheral blood samples. While this test has not been cleared or approved by the FDA, it is provided for clinical purposes in a CLIA (Clinical Laboratory Improvement Amendments)-certified laboratory.

***BCtect^®^*** (DiaGenic ASA, Norway) is a blood-based diagnostic test developed in 2008 for early detection of BC [110,111,112,117]. It uses real-time RT-PCR for expression analysis of a BC-specific 96-assay signature in whole blood, and a proprietary algorithm to distinguish between BC and non-BC patients. The signature includes genes that code for proteins involved in immune responses, signal transduction, and cellular metabolism. This test has shown efficacy for the detection of early BC in both pre- and post-menopausal women, and across cancer stages and types [110]. The assay was released as a CE IVD Mark product under the European Directive on In Vitro Diagnostic Medical Devices 98/79/EC [118], and was marketed in 20 European countries during 2009 and 2010; however, the sales fell short of the company’s expectations [119].

***Siemens Healthineers and Freenome collaborative effort.*** Recently, Siemens Healthineers and the biotech company Freenome have teamed up to develop a blood test that could improve early detection of BC and augment existing imaging technologies [120,121]. The aim is to employ Freenome’s expertise in machine learning and multiomics (epigenetic, proteomic, genomic, immunologic, and other data types) to detect early cancer, while maximising clinical accuracy. Additionally, Siemens Healthineers imaging data, as well as clinical data, will be related to molecular data to identify new BC markers complementary to those identified using current imaging [122]. In this manner, new BC biomarker identification will rely on the collaboration between multiomic and radiomic BC diagnostics.

## 8. Current Status and Future Perspectives

### 8.1. The RNA Methods Used for Breast Cancer and Their Expanding Diagnostic Potential

From the perspective of molecular diagnostics, investigations of RNAs, dynamic biomolecules involved in a variety of biological processes, have a far-reaching potential for applications in diverse areas of human health that include disease diagnosis, prognosis, and selection of therapy [123]. RNA analyses by qRT-PCR enable highly sensitive and specific detection of transcripts and quantification of low-abundance RNAs [124,125]. PCR arrays and hybridisation-based methods such as microarrays facilitate simultaneous expression profiling of multiple genes, even hundreds or thousands of genes, thus providing a more comprehensive view of the transcriptome [126]. A number of PCR-based assays are available for BC: four tissue mRNA tests (Oncotype DX, Breast Cancer Index, EndoPredict and GeneSearch BLN Test) and two blood mRNA tests (Syantra DX, BCtect). Additionally, two microarray assays (tissue-based MammaPrint, blood-based multi-cancer test Aristotle) and one nCounter-based assay (Prosigna) are offered (Table 1 and Table 2) [26,34,46,52,60,108,111]. NGS technologies represent a major advancement in RNA methods, with RNA sequencing (RNA-seq) conferring increased speed, sensitivity, and output of transcriptional profiling [127]. RNA-seq can enable the detection of a wide variety of RNA species, including mRNA, non-coding RNA, pathogen RNA, chimeric gene fusions, transcript isoforms, splice variants, and previously unidentified transcripts [123,127]. It can provide a comprehensive view of transcript abundance, but, unlike microarrays, it can also detect rare RNA transcript variants, and supports the detection of mutations and germline variation for thousands of expressed genetic variants, facilitating assessment of allele-specific expression of these variants [123,127]. The advantages offered by the NGS have led to the development of cancer diagnostic assays such as +RNAinsight, a blood test for assessing hereditary BC predisposition, and the Targeted RNA next-generation sequencing MammaPrint (i.e., an NGS variation of the MammaPrint test) [58,106]. Recent years have brought advances in cell-type specific investigations [92,93,100] and single-cell RNA sequencing (scRNA-seq) [128,129]. There is still a long way to go before scRNA-seq can be implemented in clinical practice for guiding personalised cancer treatment. However, this technique has advanced the research on the molecular mechanisms of cancer (including intratumour and intercellular heterogeneity, communication between the tumour and the immune system, clonal evolution, temporal and spatial heterogeneity, etc.), and prediction of cell sensitivities to therapy and patient prognosis [128,129].

### 8.2. The Capability of Supplemental Blood mRNA Analyses to Improve the Diagnostic Outcomes of Cancer DNA Testing

It has been well documented that RNA diagnostic testing can provide important information beyond that of DNA. This has been demonstrated by both investigational research and clinical practice [106,130,131,132]. Over the past years, a number of articles have reported that the use of transcriptomic techniques has led to a substantial improvement in diagnostic outcomes in patients whose genetic diseases could not be explained by genome or exome sequencing [133,134]. For instance, Cummings et al. reported that, by using RNA-seq, genetic diagnosis was made for 35% of subjects in a cohort of 50 patients with previously genetically undiagnosed muscle disorders [135]. It is known that standard DNA testing can miss some mutations during assessment of hereditary risk of genetic diseases or cancer [114,134,135]. However, this can be improved by transcriptional analyses. For instance, in the report by Cummings et al., RNA-seq analyses have led to the discovery of a novel highly recurrent intronic mutation causing a splice-gain event [135]. In another study, the use of whole blood RNA-seq for cases with undiagnosed rare diseases of 16 different disease categories was able to identify the causal gene and variants in 7.5% of cases [134].

Gaining extra information from mRNA analysis is also one of the important achievements in blood-level diagnostics of cancer [114,131]. Currently, Ambry Genetics is offering +RNAinsight transcriptional analyses to complement their DNA cancer panels and improve the sensitivity of hereditary risk assessment (Table 2) [106]. Blood RNA testing, performed in combination with DNA genetic testing, has been shown to identify more genetic mutations associated with increased cancer risk than DNA testing alone, and showed a 9.1% relative increase in diagnostic yield [136]. One limitation of standard DNA testing is that it can produce inconclusive results. For instance, DNA testing for cancer risk may fail to determine whether a variant increases the risk, thereby failing to give the physician enough information to recommend appropriate preventive or therapeutic steps. This can impact the patients as well as their relatives, who, as a consequence, may not be referred for genetic testing. It has been evidenced in clinical practice that blood mRNA analysis can improve the outcome of blood DNA-level genetic testing significantly by providing functional RNA information that can help identify and interpret DNA variants [18,106,113]. It can help to determine whether an uncertain DNA testing result is benign (normal) or pathogenic (disease-causing). Consequently, RNA testing can assist importantly in the management of those patients who carry pathogenic mutations but would have received negative or inconclusive results on DNA testing alone.

In addition to clinical genetic testing, where healthcare providers such as physicians, nurse practitioners, or genetic counsellors choose the appropriate test and order it from a laboratory, there has been an increasing demand from the public for direct-to-consumer (DTC) genetic tests. These are marketed directly to customers, with the customers mailing their DNA samples and receiving their results directly from the company. However, as Tandy-Connor et al. reported, a significant number of subjects who had a DTC test received a false-positive result. Consequently, the authors highlighted the importance of confirmatory testing for appropriate patient care [137]. Messenger RNA testing may represent a possibility for reliable confirmatory testing to help minimise inconclusive or even false results of both DTC and clinical DNA-genetic testing.

### 8.3. The Potential of Blood RNA Analyses to Enable Early Cancer Detection

One of the major advantages of blood mRNA analysis is its potential for early cancer detection and screening [138,139,140,141]. Since cancer prognosis depends closely on the stage of disease at diagnosis, early detection is crucial for improved patient survival. Blood mRNA tests have been developed for BC screening (Syantra DX, Syantra Inc., Calgary, NW, Canada; BCtect, DiaGenic ASA, Norway) [107,108,111] or for universal cancer screening (multi-cancer blood test Aristotle, Stage Zero Life Sciences Ltd. Richmond Hill, ON, Canada), where a single test is used for multiple cancers, including BC [116]. In BC, good performance of blood mRNA testing has been observed by Syantra DX in both pre- and post-menopausal women, as well as in women with high breast density [115]. Nonetheless, it may be worth mentioning that some of the currently offered BC and multi-cancer blood mRNA screening tests do not seem to have clearly defined populations that they are intended for, and may still be participating (e.g., Syantra DX) in ongoing clinical studies (Table 2) [106,108,109,111].

The prospects of very early cancer detection (many years before clinical diagnosis) have been brought to light by exciting recent blood biomarker research. For instance, whole blood transcriptome analysis has been shown to detect cancer-related mRNA changes up to 8 years before BC can be diagnosed with conventional approaches [65,96,97] and identify differences in blood gene expression between future metastatic and non-metastatic BC cases [64]. These results indicated that early immunological changes occurring during the carcinogenic process represent dynamic reactions to the growing cancer. Prediagnostic blood-screening possibilities have also been described for other cancers. For instance, serum RNAs have been reported to predict lung cancer up to 10 years prior to diagnosis [142,143]. Interestingly, although the samples were collected before diagnosis, RNAs measured in the samples differed, depending on the cancer staging determined at diagnosis [143]. Serum RNAs also differed between subjects with testicular germ cell tumours and healthy controls within a 10-year period prior to diagnosis [144]. Overall, blood transcriptional analyses indicate the potential for very early detection of cancer-related changes, many years before the diagnosis is possible with conventional approaches. However, the long-ranging analyses still require thorough validation, and are a while away from being adapted for cancer screening in the clinical setting.

### 8.4. The mRNA-Based Applications in Research and under Development for Personalised BC Management

In addition to early BC detection, recent research findings are also pointing to other promising future applications of blood mRNA testing to facilitate diagnosis, treatment, and prognosis. Blood mRNA analyses have been able to identify differences between lymph-node positive and negative BC [95], and between breast cancer and benign breast disease [94]. Additionally, research is ongoing for the detection of ductal carcinoma in situ [115]. Lastly, the findings of RNA sequencing of peripheral blood from BC patients suggest that new BC subtypes could be established based on mRNA levels in blood immune cells. These subtypes could potentially be used for facilitating the selection of appropriate immune therapy for individual patients [4]. Nonetheless, the utility of blood transcriptome for these applications requires extensive further experimental testing and validation in large cohorts, and certain aspects need to be addressed before these possibilities can be implemented in the clinical setting. For example, gene expression profiles of blood cells may be influenced by systemic factors such as chronic or transient inflammatory diseases or other non-cancerous diseases [93], thus necessitating patient evaluation in follow-up studies. Furthermore, the rate of false positives and false negatives needs to be examined closely. In addition, applicability to the general population needs to be considered, that includes both genders and multiple ethnicities.

### 8.5. The Issue of Centralised Testing and Restricted Availability of mRNA Diagnostics for BC

One limitation of the commercially available oncology mRNA tests is that some of them are provided as a service performed solely at the testing facilities of the companies that originally developed and validated them. Even when the samples can be collected at local designated laboratories using the prepackaged sample kits, they are then sent to a centralised laboratory for processing (e.g., multi-cancer blood test Aristotle, +RNAinsight, Syantra DX). An issue of centralised testing is that it tends to limit the clinical use of the assays [58]. For one, some countries have legal restrictions on shipping patient material outside the country. Additionally, reimbursement often requires local processing, and presents a practical obstacle to clinical implementation of these assays [58]. Decentralisation of diagnostic testing would offer several advantages. It would facilitate global availability, whereas eliminating the need for sample shipping would decrease turnaround times and costs. In addition, a direct interaction between the physician and laboratory personnel would be beneficial, especially in cases of inadequate specimens, or ambiguous or unexpected results [145]. Of course, to avoid any possibility of compromising the level of clinical validity, the assay workflow training and meticulous evaluation of concordance and reproducibility would be required between the centralised and external secondary setting [145]. Currently, none of the *blood*-based mRNA assays are offered at decentralised testing facilities. However, in recent years, efforts have been made toward the decentralisation of testing for several tissue-based BC assays, including MammaPrint and BluePrint targeted RNA next-generation sequencing assays [146], nCounter-based Prosigna [145], qRT-PCR-based Endopredict [147], and the qRT-PCR-based GeneSearch™ BLN Assay [148].

### 8.6. The Centralisation of Testing and the Associated Individualised Standardisation and Validation of RNA-Based Diagnostic Assays

Testing centralisation also means that the companies developing the tests standardise the methods for their purpose, using their own RNA protocols, including sample preparation and expression analysis, their proprietary software, and interpretation of the results. It is on the companies to ensure that the tests have been validated sufficiently according to international guidelines [149,150], since completely independent validation of these assays by other laboratories is precluded. Even the initial testing in clinical trials aimed at validating the clinical utility of these assays is carried out by the central testing facilities, with the samples sent for analysis from various locations, often from other countries [30,38,39,151,152,153]. To a certain extent, method standardisation by individual companies is inevitable. It is impossible to recommend a single experimental design for all in vitro diagnostic procedures and devices, because standardisation depends on the expression method used (e.g., microarray, NGS), the type of biological sample (e.g., FFPE, fresh tissue, whole blood, PBMCs), and the purpose of the assay, which determines the bioinformatic analysis (e.g., risk of recurrence, spreading to lymph nodes, etc.) [125,154]. However, despite the lack of common standard procedures for mRNA clinical tests, it would be beneficial to have the requirements for materials and procedures adaptable for the widest possible variety of applications and instruments [125]. Currently, there are ongoing efforts towards establishing the best practices for RNA NGS diagnostic testing, aimed at ensuring reproducibility, accuracy, and precision [123,127,149,155]. International workgroups have published guidelines for NGS diagnostics [149,150], which cover a number of topics, including quality control of specimens for multigene testing (e.g., guidelines on sample collection, processing, and storage), selection of patients for NGS testing, informed patient consent (explanation of the benefits and limitations of testing, possible disclosure of secondary findings), method implementation in a certified laboratory setting, a bioinformatics pipeline, guidelines on method validation, interpretation of the results and report preparation, communication of the results to the patient and genetic counselling, handling of acquired NGS data, and reporting of identified variants.

### 8.7. The Technical Challenges Associated with mRNA Diagnostic Testing

While DNA- and protein-level diagnostics using blood samples contribute significantly to decision-making in clinical oncology, the use of mRNA-level blood diagnostic testing has been somewhat lagging, which can be attributed to several factors. For one, acquiring adequate DNA samples is less challenging than obtaining suitable mRNA, due to the difficulties in normalisation and processing of mRNA by different laboratories [17]. Additionally, mRNA testing is more expensive. If high-throughput NGS-based investigations are used, they provide massive amounts of information, but their clinical use can be influenced by cost and reproducibility issues [156].

Nonetheless, several mRNA assays for BC are currently used successfully in the clinical setting. With regard to the processing of mRNA, there is a drawback to tissue mRNA analysis compared to blood mRNA testing; archived FFPE blocks that are used for tissue-level mRNA detection may fail the quality standard for this type of analysis due to poor preservation of the mRNA. Longer storage has been reported to impact RNA quality and the subsequent analysis of the 70-gene test, while better robustness was seen in FFPE samples that were stored for a shorter time [157]. In contrast, liquid biopsy can offer a clear advantage with regard to RNA preservation, since special tubes (e.g., PAXgene^®^ Blood RNA Tubes, BD Biosciences) can be used during blood collection to stabilise and preserve intracellular RNA immediately for further analysis, thereby yielding more accurate and reproducible gene expression data [158].

The use of RNA-protective collection tubes for blood samples is an efficient strategy to prevent damage to the RNA. Other sample stabilisation approaches include separation of blood into peripheral blood mononuclear cells or serum after collection and before freezing. However, these strategies may result in differences in the transcriptomic profiles [159]. Additionally, they may have temporal, spatial, financial, or personnel limitations [155]. Interestingly, even using different RNA preservation collection tubes (e.g., RNAgard^®^ Blood Tube or PAXgene^®^RNA Blood RNA tube) affects transcriptomic profiles, indicating the need for using a single blood collection platform during the course of a defined study [159,160]. Furthermore, it is well known that improper handling of blood samples during collection, preservation, transport, storage, and mRNA extraction can influence RNA quality and RNA sequencing results negatively [155,161]. Because of the changes that are observed in blood expression profiles due to these ex vivo influences, there is a need for reliable experimental protocols and standardised methods that would generate data that reflect the true physiological state accurately.

## 9. Conclusions

Analyses of liquid biopsy with NGS technologies have advanced biomarker discovery and personalised healthcare. They have shown utility in cancer detection, monitoring cancer evolution, and following oncological patients in real time.

The first commercial NGS sequencer, developed in 1996, was based on pyrosequencing technology [162]. Since then, a number of NGS sequencers with different chemistries have followed, and the costs of NGS sequencing has decreased progressively [127]. As the cost becomes lower, this technology will become more widely available. Continuous improvements in both high-throughput methodology, as well as bioinformatic and statistical analyses of massive amounts of transcriptome data, are expected to pave the way towards enhanced robustness and precision in cancer detection [163]. With a greater understanding of the common and distinct capabilities of DNA-level and mRNA-level testing, the utility of mRNA testing in clinical practice will increase, which will, in turn, help facilitate clinical decisions.

## Figures and Tables

**Table 2:** Commercially available blood mRNA-level diagnostic tests for breast cancer.

Assay Trade Name (Manufacturer)	Number of Genes	Assay Indicated for	Description	Methodology/ Platform	References
+RNAinsight (Ambry Genetics)	Up to 91 genes (for maximum coverage)	Assessing hereditary cancer predisposition	+RNAinsight analyses functional RNA data to classify DNA variants and identify deep-intronic mutations; intended for paired RNA/DNA analyses, as a supplement to Ambry Genetics DNA-level hereditary cancer panels CancerNext, CancerNext-Expanded, CustomNext-Cancer.	RNA sequencing	[106]
Syantra DX Breast Cancer (Syantra Inc.)	12-gene multi-biomarker panel	Breast cancer screening for women aged 25–80	Enables classification of a sample as positive or negative for BC signature; demonstrated utility for early cancer screening, for women with high breast density, and for women under 50.	qRT-PCR-based assay	[107,108]
Multi-cancer blood test Aristotle (Stage Zero Life Sciences Ltd.)	Multi-biomarker panel	Pan-cancer screening (breast, bladder, colorectum, cervix, endometrium, liver, ovary, prostate, and stomach)	Enables detection of multiple cancer molecular signatures from a single blood sample (early cancer detection).	Microarray-based assay	[109]
BCtect (DiaGenic ASA)	96-assay signature	Breast cancer screening	Enables classification of a sample as positive or negative for BC signature; utility for early BC detection in both pre- and post-menopausal women, and across cancer stages and types.	qRT-PCR-based assay	[110,111,112]

## Supplementary Materials

The following supporting information can be downloaded at: https://www.mdpi.com/article/10.3390/cancers15041087/s1, Supplementary file S1: Table S1 (List of FDA cleared or approved nucleic acid based tests for BC); Table S2 (Breast cancer therapeutics for which information for safe and efficient use is provided by the Companion Diagnostic Devices (CDDs), and a list of FDA-cleared or approved CDDs for BC).
