# Supplementary material for: Blood-Based mRNA Tests as Emerging Diagnostic Tools for Personalised Medicine in Breast Cancer
Source: Cancers (Basel). 2023 Feb 8;15(4):1087. doi: 10.3390/cancers15041087 (PMC9954278; doi:10.3390/cancers15041087)
Supplement: Supplementary file 1 [file cancers-15-01087-s001.zip › cancers-2162810-supplementary.pdf]

Supplementary file S1

Table S1. List of FDA cleared or approved nucleic acid based tests for BC [16].

Trade Name and Manufacturer	Type of test	Description	FDA reference number
23andMe PGS Genetic Health Risk Report for BRCA1/BRCA2 (Selected Variants) (23andMe)	Genetic carrier status – DNA test	Cancer predisposition risk assessment system; detection of selected BRCA1/BRCA2 variants in genomic DNA	DEN170046
Prosigna Breast Cancer Prognostic Gene Signature Assay (Veracyte, Inc.)	Multigene expression – mRNA test	Breast cancer: classifier, prognostic, recurrence risk assessment	K130010
MammaPrint (Agendia, Inc.)	Multigene expression – mRNA test	Breast cancer: classifier, prognostic, recurrence risk assessment	K101454, K081092, K080252, K070675
INFORM HER2 Dual ISH DNA Probe Cocktail (Ventana Medical Systems, Inc.)	Chromogenic In Situ Hybridization	HER2 gene status determination	P100027
HER2 CISH pharmDxTM Kit (Dako Denmark A/S)	Chromogenic In Situ Hybridization	HER2 gene status determination	P100024
HER2 Dual ISH DNA Probe Cocktail (Ventana Medical Systems, Inc.)	Chromogenic In Situ Hybridization	HER2 gene status determination	P190031
GeneSearch Breast Lymph Node (BLN) Test Kit (Veridex, LLC.)	Multigene expression – mRNA test	Sentinel lymph node, breast cancer metastasis detection	P060017 S001-S004
Dako TOP2A FISH PharmDx Kit (Dako Denmark A/S)	Fluorescence In Situ Hybridization	Topoisomerase II Alpha, Gene Amplification and Deletion	P050045 S001-S004
HER2 IQFISH PHARMDX (DAKO DENMARK A/S)	Fluorescence In Situ Hybridization	Topoisomerase II Alpha, Gene Amplification and Deletion	P040005
INSITE HER-2/NEU KIT (Biogenex Laboratories, Inc.)	Immunohistochemistry	HER2 gene status determination	P040030
SPOT-LIGHT HER2 CISH KIT (Invitrogen Corporation)	Chromogenic In Situ Hybridization	HER2 gene status determination	P050040
INFORM HER-2/NEU (Ventana Medical Systems, Inc.)	Dual In Situ Hybridization	HER2 gene status determination	P940004
DAKO HERCEPTEST (Dako Denmark A/S)	Immunohistochemistry	HER2 gene status determination	P980018

PATH VYSION HER-2 DNA PROBE KIT (Abbot Molecular, Inc.)	Fluorescence In Situ Hybridization	HER2 gene status determination	P980024
DakoCytomation Her2 FISH pharmDx™ Kit (DakoCytomation Denmark A/S)	Fluorescence In Situ Hybridization	HER2 gene status determination	P040005
Therascreen PIK3CA RGQ PCR Kit (Qiagen GMBH)	Real-time qualitative PCR test	somatic gene mutation detection in the PIK3CA gene	P190001<br>P190004

Table S2. Breast cancer therapeutics for which information for safe and efficient use is provided by the Companion Diagnostic Devices (CDDs), and list of FDA-cleared or approved CDDs for BC [16].

Therapeutics	Biomarker Description; Analyzed sample type	Companion Diagnostic Device – CDD (Manufacturer)	FDA reference number
Herceptin/ trastuzumab	HER-2 (ERBB2) gene amplification; Breast Cancer Tissue	FoundationOne CDx (Foundation Medicine, Inc.)	P170019
		HER2 CISH pharmDx Kit (Dako Denmark A/S)	P100024
		HER2 FISH pharmDx Kit (Dako Denmark A/S)	P040005
		INFORM HER2 Dual ISH DNA Probe Cocktail (Ventana Medical Systems, Inc.)	P100027
		INFORM HER-2/neu (Ventana Medical Systems, Inc.	P940004
		PathVysion HER-2 DNA Probe Kit (Abbott Molecular Inc.)	P980024
		SPOT-LIGHT HER2 CISH Kit (Life Technologies Corporation)	P050040
		Ventana HER2 Dual ISH DNA Probe Cocktail (Ventana Medical Systems, Inc.)	P190031
	HER-2 (ERBB2) protein overexpression; Breast Cancer Tissue	Bond Oracle HER2 IHC System (Leica Biosystems)	P090015
		HercepTest (Dako Denmark A/S)	P980018
		InSite Her-2/neu (CB11) Monoclonal Antibody (Biogenex Laboratories, Inc.)	P040030
		PATHWAY anti-Her2/neu (4B5) Rabbit Monoclonal Primary Antibody (Ventana Medical Systems, Inc.)	P990081
Kadcyla/	HER-2 (ERBB2) gene amplification;	FoundationOne CDx (Foundation Medicine, Inc.)	P170019

ado-trastuzumab emtansine	Breast Cancer Tissue	HER2 FISH pharmDx Kit (Dako Denmark A/S)	P040005/S009
		HER2 FISH pharmDx Kit (Dako Denmark A/S)	P040005/S009
		INFORM HER2 Dual ISH DNA Probe Cocktail (Ventana Medical Systems, Inc.)	P100027/S030
	HER-2 (ERBB2) protein overexpression;<br>Breast Cancer Tissue	HercepTest (Dako Denmark A/S)	P980018/S016
		PATHWAY anti-Her2/neu (4B5) Rabbit Monoclonal Primary Antibody (Ventana Medical Systems, Inc.)	P990081/S039<br>513(f)
Perjeta/<br>pertuzumab	HER-2 (ERBB2) gene amplification;<br>Breast Cancer Tissue	FoundationOne CDx (Foundation Medicine, Inc.)	P170019
		HER2 FISH pharmDx Kit (Dako Denmark A/S)	P040005/S006
	HER-2 (ERBB2) protein overexpression;<br>Breast Cancer Tissue	HercepTest (Dako Denmark A/S)	P980018/S015
Lynparza/<br>olaparib	BRCA1 and BRCA2 mutations;<br>Breast Cancer Whole Blood	BRACAnalysis CDx (Myriad Genetic Laboratories, Inc.)	P140020/S012
	PIK3CA, C420R, E542K, E545A, E545D [1635G>T only], E545G, E545K, Q546E, Q546R, H1047L, H1047R, and H1047Y;<br>Breast Cancer Tissue	FoundationOne CDx (Foundation Medicine, Inc.)	P170019/S006
Talzenna/<br>talazoparib	BRCA1 and BRCA2 Mutations;<br>Breast Cancer Whole Blood	BRACAnalysis CDx (Myriad Genetic Laboratories, Inc.)	P140020/S015
Piqray/<br>alpelisib	PIK3CA; C420R, E542K, E545A, E545D [1635G>T only], E545G, E545K, Q546E, Q546R, H1047L, H1047R, and H1047Y;<br>Breast Cancer Plasma (FoundationOne liquid CDx) and<br>Breast Cancer Tissue or plasma (Therascreen PIK3CA RGQ PCR Kit)	FoundationOne Liquid CDx (Foundation Medicine, Inc.)	P200006
		therascreen PIK3CA RGQ PCR Kit (QIAGEN GmbH)	P190001<br>P190004
Verzenio/<br>abemaciclib	Ki-67 protein expression;<br>Breast Cancer Tissue	Ki-67 IHC MIB-1 pharmDx (Dako Omnis) (Agilent Technologies)	P210026
Keytruda/<br>pembrolizumab	PD-L1 protein expression;<br>Triple-Negative Breast Cancer Tissue	PD-L1 IHC 22C3 pharmDx (Dako North America, Inc.)	P150013/S020
